# Blood spots screening for identification of Fragile X Syndrome among intellectual disability students in Flores Island, INDONESIA

**DOI:** 10.1186/1687-9856-2013-S1-P204

**Published:** 2013-10-03

**Authors:** Agustini Utari, Joyce Lo, Tzuhan Tong, Tri Indah Winarni, Sultana MH Faradz, Flora Tassone

**Affiliations:** 1 Division of Human Genetics, Center for Biomedical Research, Faculty of Medicine, Diponegoro University, Semarang, Indonesia; 2 MIND Institute, University California Davis, Sacramento, California,USA; 3 Department of Biochemistry and Molecular Medicine, School of Medicine, University California Davis, Sacramento, California, USA; 4 Department of Pediatrics, Faculty of Medicine, Diponegoro University, Indonesia

## 

Fragile X Syndrome (FXS) is the most common known inherited form of intellectual dissability (ID), caused by a CGG repeat expansion located in 5’ untranslated region of the *FMR1* gene. The prevalence for both males and females varies in different populations being about 1 in 2600-4000 for the full mutation and 1 in 130-800 for premutation alleles. Previous screening in Indonesia showed FXS prevalence of 1.9%(Faradz *et al.* 1999). Advances targeted treatments in FXS have led to a newborn and high risk populations FXS screening studies. In this study, a rapid and inexpensive method for screening both males and females for *FMR1* allele sizes throughout the premutation and full-mutation range using a dried blood spot, (Tassone et al., 2008) was applied for the screening of FXS in Flores Island, one of the very remote area in East Indonesia.

The screening includes 211 children (130 males and 81 females) from an institution and school for children with special needs in Flores Island, East Nusa Tenggara, Indonesia.

The Blood spots PCR analysis result showed the presence of 3 expanded alleles (1.42%) consist of 2 males with a full mutation and 1 male with a premutation allele. Southern blot anlysis confirmed the presence of a full mutation allele and determined the methylation status in this individual.

Our results suggest that blood spots screening is an inexpensive and simple method to perform high premutation *FMR1* in high risk population especially in the remote area that are far from laboratory facilities.

